# Contemplating or Acting? Which Immersive Modes Should Be Favored in Virtual Reality During Physiotherapy for Breast Cancer Rehabilitation

**DOI:** 10.3389/fpsyg.2021.631186

**Published:** 2021-04-08

**Authors:** Hélène Buche, Aude Michel, Christina Piccoli, Nathalie Blanc

**Affiliations:** ^1^Laboratoire Epsylon EA 4556, Université Paul Valéry, Montpellier III, Montpellier, France; ^2^Montpellier Institut du Sein, Clinique Clémentville, Montpellier, France; ^3^Kinesitherapeute, Montpellier Institut du Sein, Clinique Clémentville, Montpellier, France

**Keywords:** virtual reality, breast cancer, immersion, anxiety, distraction, physiotherapy

## Abstract

**Background:**

Even though virtual reality (VR) is more and more considered for its power of distraction in different medical contexts, the optimal conditions for its use still have to be determined in order to design interfaces adapted to therapeutic support in oncology.

**Objective:**

The objective of this study was to examine the benefits of VR using two immersion methods (i.e., one participatory, one contemplative) and comparing them with each other in a population of women with breast cancer who have undergone breast surgery, during scar massage sessions.

**Methods:**

In a physiotherapy center, each patient participated in four experimental conditions in a random order: two sessions used virtual immersion (i.e., one participatory and one contemplative), one session proposed musical listening and the fourth one was a standard session care. The impact of the level of patient involvement in the virtual world was apprehended through the evaluation of the feeling of presence; the estimation of elapsed time of the physiotherapy sessions and particular attention was paid to the evaluation of patient emotional state.

**Results:**

Our study showed an increase in positive emotions (i.e., joy and happiness) and a decrease in anxiety regardless which support methods were offered. Participatory VR created a feeling of more intense spatial presence.

**Conclusion:**

Our results highlight the importance of the context in which VR should be offered. The presence of the practitioner and his interactions with the patient can provide a context just as favorable in reducing anxiety as the emotional regulation tools used (VR, music). The use of technological tools should be favored when the practitioner is unavailable during the treatment phase or, even, in order to reduce the monotonous nature of repetitive therapeutic sessions.

## Introduction

Breast cancer is considered the most common form of cancer in women and represents 16% of all cancers in women (WHO). Over 1.3 million women worldwide are diagnosed with breast cancer each year (Zhang et al., 2019). While the incidence of this cancer has significantly increased in recent years, its survival rate has nevertheless improved considerably (Loh and Musa, 2015). Despite this positive evolution, the diagnosis of cancer and the associated treatments, as well as the more or less long-term aftereffects, are still particularly stressful (Arrieta et al., 2013). Approximately 55% of patients treated for cancer have been shown to have clinical symptoms of anxiety disorder (O’Connor et al., 2010) and/or depression (Harrington et al., 2010; Hill et al., 2011). The importance of psychological care to relieve cancer patients and, even more, the impact of this care on their compliance with treatment has been widely demonstrated (Lutgendorf et al., 2010). Beyond conventional psychotherapies and psycho-oncology supportive therapies, various stimulations such as music (Gramaglia et al., 2019), Tai Chi (Wayne et al., 2018) or yoga (Lin et al., 2018) have been proposed and found to be effective in reducing the most common side effects (i.e., tiredness, anxiety, depression, sleep disorders; Chirico et al., 2019; Maindet et al., 2019).

In recent years, virtual reality (VR) has gradually established itself in the medical field thanks to new technological advances allowing more and more immersive and efficient entertainment devices. Concretely, virtual reality places the user in an artificial world where everything around him, including his physical environment, is replaced by images and sounds entirely generated by computer. Thus, totally freed from the contingencies of reality, the patient finds himself projected into an artificial universe providing different kinds of sensory stimulations in which his senses can be stimulated simultaneously. The effectiveness of VR comes from the intensity of this multisensory immersion, known as the feeling of presence (i.e., subjective experience of being in one place or one environment, even when you are physically in another one; Witmer and Singer, 1998; Indovina et al., 2018; Tennant et al., 2020).

The benefits of VR were assessed for the first time in oncology in chemotherapy sessions with very promising results (Oyama et al., 1999; Schneider and Workman, 1999; Schneider et al., 2003, 2004). Since then, many studies have focused on the effectiveness of VR in improving patient management during cancer treatment (Chirico et al., 2016; Chow et al., 2020). Most of the work in oncology tends to show that the use of VR during chemotherapy reduces the most common side effects (i.e., vomiting, pain, tiredness) and promotes a decrease in anxiety as well as an improvement in mood (Chirico et al., 2016; Bani Mohammad and Ahmad, 2018; Sharifpour et al., 2020). In addition, thanks to its power of distraction, VR also allows the perception of time to be modified: patients in chemotherapy systematically underestimate the duration of treatment (Schneider et al., 2011). In parallel, studies have been developed in oncology to face pain during painful procedures (i.e., venipuncture, port access). In the majority of cases, there was a significant reduction in perceived pain (Gershon et al., 2003; Wolitzky et al., 2005; Windich-Biermeier et al., 2007; Nilsson et al., 2009; Atzori et al., 2018; Gerçeker et al., 2020; Semerci et al., 2020). Patients who are distracted using VR have also shown an increase in relaxation and feelings of peace and are significantly less frustrated during the oncology painful procedures (Scates et al., 2020). If few studies have reported the same advantage in reducing chronic cancer pain by using VR at home (Garrett et al., 2020), numerous studies have documented the relevance of using VR in the context of patient hospitalization. Its use can relieve pain and anxiety in hospital patients with breast cancer (Bani Mohammad and Ahmad, 2018) as well as in younger patients hospitalized in oncology (Tennant et al., 2020). Recent studies have extended the benefits of VR to radiation therapy sessions (Marquess et al., 2017; Chirico et al., 2019; Ahmad et al., 2020) and to palliative care (Niki et al., 2019; Johnson et al., 2020).

Most of the work examining the effectiveness of VR has focused on an acute phase of cancer care (Chirico et al., 2016; Zeng et al., 2019; Ahmad et al., 2020; Chow et al., 2020). Few studies have been carried out during a cancer rehabilitation phase. To date, no research has evaluated the impact of VR as a tool for relaxation during physiotherapy rehabilitation following breast cancer. However, following treatment for breast cancer (surgery, lymph node removal, axillary radiotherapy), physical complications such as stiffness in shoulder mobility, lymphedema of the upper limb or pain associated with scars are frequent (Stubblefield and Keole, 2014). They can have a damaging impact on the quality of life of patients with sometimes considerable physical, social and psychological repercussions (Weiss and Spray, 2002). Complications with anxiety and depression have also been observed (Schreiber et al., 2014). Therefore, it is understood that these patients must benefit from appropriate physiotherapy in rehabilitation, which is now well identified (Rafn et al., 2018). Virtual reality, thanks to its distraction and relaxation capacities, should make it possible to facilitate these physiotherapy sessions. The use of VR-based therapies could be a successful strategy to improve the tolerance of post-operative physiotherapy care. Feyzioǧlu et al. (2020) recently reported that it improved motor functionality.

The distracting power of VR represents a real asset in oncology. By visually isolating the patient from the medical context, it allows the individual’s attention to focus on the virtual experience and be distracted from the unpleasant stimuli of the stressful environment (Zeng et al., 2019). Therefore, the distraction should induce positive valence emotions, reduce the level of anxiety and lead to an underestimation of the duration of treatment (Schneider et al., 2011; Chirico et al., 2019). Several studies have shown how high-quality, technological and interactive VR devices enhance the benefits of the power of distraction (Hoffman et al., 2006; Indovina et al., 2018; Chirico et al., 2019). One of the key factors underlying the distracting power of VR is its multisensory and interactive aspect allowing people to be involved in the virtual world (Indovina et al., 2018; Chirico et al., 2019; Ahmadpour et al., 2020). This involvement in the immersive task, understood as the interactive potentials allowing it to act on the environment (e.g., move around, make certain elements appear, solve a cognitive task, etc.), is correlated with the intensity of the feeling of presence guarantor of VR efficiency (Birnie et al., 2018; Ahmadpour et al., 2020). The quality of the immersion, the degree of interaction and the involvement of the individual would therefore be crucial parameters impacting the effectiveness of distraction under VR.

Several studies have tested the effectiveness of interactive VR in oncology by engaging patients in the virtual environment with varying degrees of involvement. Certain tasks included making choices and decisions in order to solve a riddle (Schneider et al., 2003, 2004, 2011; Schneider and Hood, 2007). Others proposed to paint 3D illustrations by simple hand and arm movements (Higgins et al., 2019). The majority of these studies chose applications requiring minimal interactions (i.e., pointing and clicking with the device’s remote control). Only one study have proposed space travel with the possibility of launching rockets on targets such as planets (Johnson et al., 2020), while others have favored exploration in a relaxing natural universe (Chirico et al., 2019) by offering the possibility of shaping the environment as desired, like changing the color of flowers (Li et al., 2016). These aimed to engage the patient’s attention on objects or characters contained in a particular virtual environment.

Today, if the quality of immersion is clearly correlated with the quality of the high-tech system used (Indovina et al., 2018; Chirico et al., 2019), no study so far has compared the effectiveness of different degrees of interaction with the involvement of patients under VR. To the best of our knowledge, only one study has evaluated the effectiveness of interactive VR during chemotherapy by comparing its effects with music therapy, without comparing the results to those of passive immersion (Chirico et al., 2019). In that study, one sample of 94 women with breast cancer was randomly assigned to one of the three following conditions: the VR condition, the music condition and a control condition (i.e., standard chemotherapy). Patients in interactive VR condition moved around in a natural environment (e.g., island, forest, mountain, sea) while patients in music condition listened to relaxing music. According to measurements of anxiety (STAI; Spielberger et al., 1983) and mood states (SVPOMS; Shacham, 1983), Chirico et al. (2019) reported that anxiety, depression and tiredness were reduced more with interactive VR than with music therapy. However, both of these types of intervention proved to be beneficial compared to the control group who was subjected to chemotherapy under conventional conditions.

Following the work of these authors, the objective of our study was to compare the effectiveness of different VR apparatuses (i.e., contemplative VR vs. participatory VR) as distractive tools for patients with breast cancer in a rehabilitation phase (post-surgical physiotherapy care), these VR apparatuses being compared to music listening condition. To achieve this, our goal was to conduct this comparison by allowing patients to experience the different immersive modalities of VR as well as the listening music condition. In line with Chirico et al. (2019), we assumed that if the patient engagement is reinforced under participatory VR, then we should observe better quality of immersion leading to a more marked benefit in terms of feelings (emotional and temporal) during this classical scars massage session. In other words, we hypothesized that the more the engagement of the patients, the more efficient is the distractive tool.

## Materials and Methods

### Sample

The participants were recruited from a physiotherapy center that is associated with the Clémentville Clinic Oncology Department, the MIS (Montpellier Institut du Sein) located in Montpellier. The inclusion criteria were as follows: (1) be monitored for non-metastasized breast cancer; (2) not be in a phase of cancer recurrence; (3) have had breast surgery; (4) receive physiotherapy rehabilitation care namely, scar massage sessions; (5) know how to read and write in French; (6) be over 18 years old; (7) Patients wearing glasses were included in the study because the VR headset had an adapter provided for this purpose. To prevent the risk of discomfort associated with the VR, patients with vestibular disorders or having reported a history of motion sickness were excluded. In addition, the presence of epileptic disorders, alcohol or drug addiction were also clinical exclusion factors from the study.

### Materials

Our study aimed at comparing the effectiveness of various distractive interventions (music listening vs. VR) among patients undergoing treatment for breast cancer. As a continuation of the work conducted by Chirico et al. (2019), the present study examined the impact of different kinds of virtual stimulation (i.e., contemplative vs. participatory). Given the appeal of patients suffering from breast cancer to natural environments (Michel et al., 2019), *Greener Gamer’s Nature Treks VR* relaxation application (Carline and Carline, 2017) was selected. This application has nine relaxing visual environments with relaxing sounds. The particular interest of *Nature Treks VR* is due to its two immersive modes: one contemplative, the other participatory. In the participatory version, in addition to contemplative exploration, the patient is invited, using specific joysticks, to shape her own environment (e.g., control the weather, plant trees or flowers, spawn animals.).

For the music listening condition, “Spring” from Vivaldi’s Four Seasons was selected. A specific extract was chosen for its well-known effectiveness in inducing a positive emotional state (see Krumhansl, 1997).

A booklet was created to allow the follow-up of each patient’s responses on the four experimental conditions. All booklets began with the presentation of the study, followed by the letter of consent and a demographic questionnaire. The booklet also included all the questionnaires used during the different stages of this study with the exception of the ones that aimed to assess their anxiety (i.e., *STAI-YB*) and their immersion capacity (i.e., *QPI Propension à l’immersion*). These were proposed at a later stage.

#### Assessment of the Feeling of Presence and the Feeling of Elapsed Time

The Independent Television Commission–Sense of Presence Inventory (ITC–SOPI, 2000) inspired by the original version of: Witmer and Singer (1998) was used to assess the feeling of presence and immersion in the virtual environment. The *ITC–SOPI* included 44 questions to assess four sub-factors: the spatial presence felt inside the VR device, the engagement indicating the level of user involvement in the immersive task, the natural aspect of the environment and the negative effects that may be generated by the apparatus. Responses were collected using a scale from 1 (strongly disagree) to 5 (strongly agree).

In order to estimate the elapsed time, a visual analogic scale ranging from 0 to 40 min regulated every 5 min was presented after each scar massage session.

#### Measurement of Mood States

Like Chirico et al. (2019), we assessed the emotional state of patients. However, we opted for using the *Self-Asssessment Manikin (SAM)* scale by Bradley and Lang (1994), regarding its usability in clinical context (physiotherapy session). The *SAM* is a pictorial (non-verbal) evaluation technique that enabled to measure patients’ emotional responses under the four conditions of the study. The emotional response was evaluated regarding contentment (1: not happy to 9: happy) and arousal level (1: calm to 9: excited).

#### Anxiety Measurement

For the purposes of this study, we chose to exploit two measures of anxiety level. Situational anxiety was assessed using *the State Anxiety Inventory* (*SAI*) *for adults* (Spielberger et al., 1983), which is a valid and commonly used tool for measuring anxiety. The *STAI-YA* self-assessment scale was also used by Chirico et al. (2019). It assesses the subjective feelings of apprehension felt “in the moment, just at this moment” toward an aversive or therapeutic situation (Spielberger et al., 1983). The scale is made up of 20 items coded in four points (no, rather no, rather yes, and yes).

Anxiety was also assessed using the *Anxiety-Trait Inventory* (Spielberger et al., 1983). The *STAI-YB* Self-Report Scale was used to ensure that the anxiety levels observed did not reflect the anxiety usually experienced by patients. This control scale is coded according to four points (almost never, sometimes, often, and almost always).

#### Evaluation of Cybersickness Symptoms

The evaluation of side effects (nausea, headache, and dizziness, etc.) that may be caused by VR was carried out using a *questionnaire on cybersickness (QC)* from the Cyberpsychology Laboratory of UQO (2002). The *QC* consists of 16 items measuring the intensity of cybersickness in four points (0: not at all to 3: severely). The version used was a French Canadian translation (Kennedy et al., 1993).

#### Experiment Feedback

A writing space was provided at the end of the booklet to collect feedback on patient experience. The participants were invited to answer several questions to know their opinions on the four sessions in which they had participated. The first question (Q1), via a nine-point scale ranging from 1 for “not at all” to 9 for “very”, was to determine if patients were supportive of the use of VR. The second question (Q2), a Yes/No question, was about the physical inconvenience resulting from the immersion. The third question (Q3), also a Yes/No question, assessed the feeling of losing track of time during the immersion. Then, the contributions of VR were evaluated using a six-point scale going from 0 for “not at all” to 5 “completely”, specifying the possibilities of escaping, of being distracted, of better accepting care, of feeling positive emotions or of reducing negative emotions (Q4). They were then asked to indicate their immersion preference, namely “contemplative” or “active” (Q5), and to decide whether the participatory immersion offered sufficient possibilities for interaction (Q6). Patients were also invited to specify whether or not they would like VR sessions in the form of evolving scenarios at each immersion (Q7). Finally, they were asked, using a nine-point scale ranging from 1 for “not at all” to 9 for “completely”, whether they would recommend the use of VR during physiotherapy sessions to people with cancer (Q8).

### Apparatus

An *Oculus Go*^®^ headset consisting of an integrated 5.5-inch screen with a resolution of 2560 × 1440 pixels and a 110° field of view was used. The headset included a remote control that allowed navigation in the virtual world and an accessory allowing the helmet to be worn with glasses. This device, perfectly suited for use in a medical environment, had the advantage of being completely autonomous. Unlike other VR systems, it did not require the use of a computer or game console. Its 6 GB of RAM and 64 GB of storage allowed direct access to games.

For the music listening condition, we used an audio headset with the following characteristics: *Hi-Fi Beats by DR.DRE*^®^ *SOLO HD*, an on-ear model that includes two speakers in each earpiece. The headset was plugged into a *smartphone SAMSUNG S9*^®^.

### Experimental Design and Procedure

Based on the study by Chirico et al. (2019), we adapted the protocol to a different stage of breast cancer management: the physiotherapy rehabilitation after surgery. We wanted to evaluate in an intra-individual design, the benefits of the different distraction techniques. Like Chirico et al. (2019), we compared the effects of music listening and VR. However, in order to go one step beyond this study, VR was offered in two modes of immersion involving different degrees of attention: one purely contemplative and the other participatory. All three distractions were compared to a distraction-free control condition. Therefore, each patient experienced the four test conditions (i.e., music listening, contemplative VR, participatory VR, and classical scar massage session).

At the beginning of the experiment, all of the patients had already completed several physiotherapy sessions during which they were able to develop a relationship with the same practitioner who worked on positive support, with empathy and closeness. The control condition was that for which no recourse to a specific entertainment tool (VR or music listening) replaced the positive support of the practitioner.

The sessions were carried out over a period of 10 months in a physiotherapy center, mostly from March 2019 to January 2020. Participants who met the inclusion criteria were approached during one of their visits to the physiotherapy center that endorsed data anonymization. The experimenter presented the ins and outs of the study to patients and left them a week of reflection. During the week of reflection, the experimenter was available to answer their questions by phone or email, but no patient asked questions about the study. In order to guarantee the same human context for all patients, the same practitioner from the center was asked to perform all the sessions. The patients experienced the four types of physiotherapy sessions (i.e., participatory VR, contemplative VR, music listening, and classical scar massage session) in random order. The individual sessions lasted approximately 30 min.

All the patients agreed to participate in this study by signing an informed consent form specifying the general context and the different stages of the research. Because the survey was low risk, the participants had consented, and the data were not of a sensitive nature or confidential and were completely anonymous with no personal information being collected, no formal ethical review was required. All study data were stored in a secure location and were not intended to be disseminated to other researchers. Once the patients signed the consent form, they were then invited to complete the demographic questionnaire. Once they were set up individually in their treatment room, patients were systematically informed of the instructions relating to the support they would receive during the session before completing the first series of questionnaires (i.e., *SAM and STAI-YA*).

During the four physiotherapy sessions, the patients lay on a tilt treatment table. The physiotherapist provided the scar massage of the breast. Each session lasted an average of 30 min. The distraction intervention (i.e., participatory VR, contemplative VR, and music listening) always took place at the beginning of the physiotherapy session after having received the instructions for the various distractions and having completed the first series of questionnaires.

For physiotherapy treatments with VR, the experimenter began by detailing the different environments so that each participant could select her universe. The experimenter then showed her how to use the VR equipment according to the assigned condition. The participatory and contemplative immersions consisted of walking in the selected universe without having to physically move. In addition to exploring, active immersion allowed weather control, night or day and planting trees or flowers to shape her own environment. The experimenter helped the participants put on the VR headset and program the desired environment for direct access to relaxation. The chosen virtual environment was identical during the two immersions and the participants used the equipment for 10 min during the two sessions. At the end of the immersion, each participant was invited to complete the last series of questionnaires (i.e., *SAM*, *time estimation*, *STAI-YA*, and *ITC-SOPI*) and the control questionnaires (i.e., *STAI-YB and QC cybersickness*).

For the music listening physiotherapy session, the experimenter prepared the music using a smartphone before helping the participants put on the headphones. Patients listened to the music for 10 min. After listening, each participant was invited to complete the last set of questionnaires (i.e., *SAM, time estimation, and STAI-YA*).

For the classical scar massage session, participants completed the first set of questionnaires (i.e., *SAM and STAI-YA*) at the beginning of their session and the last set of questionnaires (i.e., *SAM, time estimation, and STAI-YA*) at the end of their session.

At the end of the study, after their fourth session, the participants were asked to answer a multiple choice questionnaire by checking the boxes that best corresponded to their VR experiences.

## Results

Our sample consisted of 52 patients with breast cancer. Each patient experienced the four proposed conditions in random order. These patients were between 28 and 77 years old (average age = 56.02 years ± 10.62). Out of the 52 patients recruited, 46 participated in all the experimental conditions of the study. Two patients did not continue the study, one for lack of interest, and the second due to difficulties after the first VR session (she felt dizzy 1 h after the immersive task). Four patients did not return for their physiotherapy sessions. Therefore, we treated 88.46% of the initial number of participants. The socio-demographic characteristics of the participants are presented in Table 1.

**TABLE 1:** Baseline data of participants.

Variables	Participants	%
Age: Mean (SD)	56.02	
	(10.62)	
**Marital status**		
Married	*N* = 28	60.87
Single/widowed/divorced	*N* = 18	39.13
**Employment**		
Yes	*N* = 21	45.65
No	*N* = 25	54.35

The JASP software was used to perform the statistical analyzes. We chose to focus our attention on the following measurements: the feeling of presence and the perception of time in VR conditions (participatory VR and contemplative VR), the induction of positive emotions (valence and arousal) as well as anxiety. To test our hypotheses, repeated measures ANOVAs were calculated similar to those performed in related studies (Schneider and Workman, 1999; Gershon et al., 2004; Hoffman et al., 2006; Chirico et al., 2019). Like previous works assessing the VR efficacy, paired Student’s *t*-tests were performed when indicated, to reveal differences between modalities. Regarding patients’ anxiety, responses to the trait anxiety control questionnaires were compared to the standards with a simple Student’s *t*-test. The threshold of 0.05 was adopted for all statistical analyzes. The holm correction was applied for all statistics.

### Feeling of Presence and Perception of Time

#### Feeling of Presence

We wanted to determine the impact of the nature of the immersion (participatory vs. contemplative) on the induction of the feeling of presence. The mean differences collected using *ITC SOPI* were compared by factors, namely spatial presence, engagement, naturalness of the environment and negative effects (see Table 2). We performed four *ANOVAs* (one per factor according to the expert’s recommendations for this scale) with a factor with immersion (participatory VR vs. contemplative VR) as the intra-participant factor.

**TABLE 2:** Means and standard deviation of the sense of presence by presence factor according to the condition.

	Participatory VR	Contemplative VR
	Mean (SD)	Mean (SD)
Spatial presence	3.65 (0.74)	3.14 (0.78)
Engagement	3.83 (0.67)	3.78 (0.75)
Nature of environment	3.70 (1.05)	3.54 (1.15)
Side effects	1.44 (0.66)	1.51 (0.78)

*SD, standard deviation. Spatial presence participatory VR vs. contemplative VR: *p* < 0.001.*

The variance analysis revealed an effect of the virtual immersion modality on spatial presence, *F* (1,45) = 12.46; *p* < 0.001, η^2^*p* = 0.217. More precisely, the spatial presence of the participants was higher during the participatory immersion (*M* = 3.65, SD = 0.74) than during the contemplative immersion (*M* = 3.14, SD = 0.78). In accordance with our hypothesis, participatory VR induced a more intense feeling of presence in patients.

In contrast, the patient’s engagement in the virtual environment was identical during the two immersions *F* (1,45) = 0.14; *p* = 0.71, η^2^*p* = 0.003. Thus, the participants were no more engaged in actively navigating the environment (*M* = 3.83, SD = 0.67) than in passively contemplating it (*M* = 3.78, SD = 0.75). It appears that 45.65% of patients (i.e., 21 out of 46) considered that the “active” mode was not sufficiently interactive. The engagement results could be explained by the lack of interactive possibilities offered by the virtual environment. Similarly, 67.39% of patients would have liked to have been offered VR sessions in the form of evolving scenarios (31 out of 46 participants).

Similarly, the virtual immersion modality had no effect on the natural aspect of the environment, *F* (1,45) = 0.62; *p* = 0.34, η^2^*p* = 0.020. The environment did not appear significantly more natural during the participatory immersion (*M* = 3.7, SD = 1.05) than during the contemplative immersion (*M* = 3.54, SD = 1.15).

#### Time Perception

Lastly, we wanted to assess to what extent the apparatus could reduce the estimated time of physiotherapy sessions. To do this, we examined the differences between real time and perceived time depending on the conditions (see Table 3). We performed a type III two-factor repeated measures *ANOVA* (conditions, participatory VR vs. contemplative VR vs. music listening vs. classical massage session) × 2 (Time, real vs. estimated).

**TABLE 3:** Means and standard deviation of time (real vs. estimated) by experimental condition.

	Real time	Estimated time
	Mean (SD)	Mean (SD)
Participatory VR	30 (0)	12.91 (6.31)
Contemplative VR	30 (0)	11.37 (5.46)
Music listening	30 (0)	21.91 (11.95)
Classical scar massage session	30 (0)	27.33 (7)

*SD, standard deviation. Real time vs. Estimated time: *p* < 0.001.*

The variance analysis revealed a main effect of time (actual vs. estimated) *F* (1,45) = 249.65; *p* < 0.001, η^2^*p* = 0.847. The patients estimated the elapsed time of the sessions to be shorter (*M* = 18.38, SD = 10.36) than the real time (*M* = 30, SD = 0). There was also a main effect of the condition *F* (3,135) = 48.75; *p* < 0.001, η^2^*p* = 0.52 and an interaction between time and condition *F* (3,135) = 48.75; *p* < 0.001, η^2^*p* = 0.52. The proposed distractions did have an effect on the temporal perception of the patients. Namely, the *t*-test showed that the differences between the estimated time with participatory VR and the estimated time with contemplative VR were not significant [*t* (45) = 1.74, *p* = 0.089]. The participatory immersion (*M* = 12.91, SD = 6.31) did not reduce the estimated session time any more than the contemplative immersion (*M* = 11.37, SD = 5.46).

However, the time perceived by the patients during the physiotherapy session was significantly shorter when they were under participatory VR than when they listened to music *t* (45) = −5.155, *p* < 0.001 or when they did not have any distractions *t* (45) = −11.465, *p* < 0.001.

With contemplative VR, the time perceived by patients during the physiotherapy session was also significantly shorter when they were under contemplative VR than when listening to music *t* (45) = −5.433, *p* < 0.001 or when they had no distractions at all *t* (45) = −12.711, *p* < 0.001.

Finally, the elapsed time perceived by the patients during the physiotherapy session was significantly shorter when listening to music than during their classical scar massage session *t* (45) = −2.91, *p* = 0.05.

To conclude, the three distractions made it easier to underestimate the elapsed time for physiotherapy sessions. However, the two virtual immersions were more effective than music in reducing the perceived time of the sessions. Therefore, it seems that patients feel they lose track of time during their journey through the virtual universe, regardless of their involvement in it.

### Mood States

For the induction of emotions measurement, we observed differences in the valence scores (see Table 4) and arousal (see Table 5) on the *SAM* scale. We calculated two repeated measures, type III two-factor *ANOVA*s (Condition, participatory VR vs. contemplative VR vs. music listening vs. classical scar massage session) × 2 (Time of measurement, before vs. after) on the valence and on emotional arousal separately considered.

**TABLE 4:** Means and standard deviation of emotional valence by experimental condition as a function of the time of measurement.

	Before	After
	Mean (SD)	Mean (SD)
Participatory VR	6.91 (2.07)	7.87 (1.44)
Contemplative VR	7.15 (2.09)	8.52 (1.05)
Music listening	7.13 (1.87)	8.26 (1.08)
Classical scar massage session	7.24 (1.84)	8.04 (1.35)

*SD, standard deviation. Valence before vs. after *p* < 0.001.*

**TABLE 5:** Means and standard deviation of emotional arousal by experimental condition as a function of the time of measurement.

	Before	After
	Mean (SD)	Mean (SD)
Participatory VR	4.11 (2.56)	2.13 (1.54)
Contemplative VR	3.89 (2.42)	2.15 (1.76)
Music listening	3.39 (2.29)	2.21 (1.69)
Classical scar massage session	3.02 (2.08)	2.26 (1.69)

*SD, standard deviation. Arousal before vs. after *p* < 0.001.*

Regarding the emotional valence measure, we obtained a time effect *F* (1,45) = 60.92; *p* < 0.001, η^2^*p* = 0.575. The emotional feeling was more positive (i.e., participants were much happier) after the experience (*M* = 8.17, SD = 1.26) than before (*M* = 7.11, SD = 1.96). The variance analysis did not indicate an effect on the condition of emotional feeling *F* (3,135) = 1.05; *p* = 0.37, η^2^*p* = 0.023, or an interaction between the moment of measurement and the condition *F* (3,135) = 0.96; *p* = 0.416, η^2^*p* = 0.021.

If the *ANOVA* did not detect any differences between the conditions, based on the *t*-test, we found a significant difference between the emotional valence means after participatory VR and after contemplative VR *t* (45) = −2.798, *p* = 0.008. Thus, the patients appeared significantly more joyful after the session under contemplative immersion (*M* = 8.52, SD = 1.05) than after the session under participatory immersion (*M* = 7.87, SD = 1.44).

Regarding emotional arousal, the results again revealed a main effect on the time of the measurement *F* (1,45) = 79.25; *p* < 0.001, η^2^*p* = 0.638. The emotional arousal was higher before the experiment (*M* = 3.60, SD = 2.36) than after it (*M* = 2.19, SD = 1.66). As the *SAM* scale associates the highest value with the adjective “excited” and the lowest with the adjective “calm”, these results reflect a calming effect. We observed no effect on the condition of emotional arousal *F* (3,135) = 0.96; *p* = 0.414, η^2^*p* = 0.021. No matter the offered support (participatory VR, contemplative VR, music listening or classical scar massage session), we found an increase in positive emotional state and a decrease in negative emotional arousal after each session.

We observed an interaction between the moment of measurement and the condition *F* (3,135) = 3.57; *p* = 0.016, η^2^*p* = 0.074. From the *t*-test, the results indicated significant differences between participatory VR and the classical scar massage session *t* (45) = 2.437, *p* = 0.019 only before the experiment. The patients had a higher level of arousal before having the participatory immersion experience (*M* = 4.11, SD = 2.56) than before their classical scar massage session (*M* = 3.02, SD = 2.08). We also note significant differences between the contemplative VR and the classical scar massage session *t* (45) = 2.082, *p* = 0.043. The patients also had a higher level of arousal before experiencing contemplative VR (*M* = 3.89, SD = 2.42) than before their classical scar massage session (*M* = 3.02, SD = 2.08).

### Anxiety

To report the effect of anxiety reduction, the data was encoded and transformed according to the guidelines of the *STAI-YA* standard. We observed differences in the anxiety means reported on the *STAI-YA* scale (see Table 6). We used a type III two-factor repeated measures *ANOVA* (condition, participatory VR vs. contemplative VR vs. music listening vs. classical scar massage session) × 2 (time of measurement, before vs. after) on the anxiety state.

**TABLE 6:** Means and standard deviation of anxiety by experimental condition as a function of the time of measurement.

	Before	After
	Mean (SD)	Mean (SD)
Participatory VR	35.52 (15.12)	27.02 (8.10)
Contemplative VR	35 (12.59)	26.22 (6.41)
Music listening	33.26 (11.27)	28.41 (8.95)
Classical scar massage session	33.54 (10.95)	28.83 (9.59)

*SD, standard deviation. Anxiety before vs. after *p* < 0.001.*

We observed a main effect at the time of measurement *F* (1,45) = 60.55; *p* < 0.001, η^2^*p* = 0.574. Patients’ anxiety was lower after the experiments (*M* = 27.62 SD = 8.35) than before them (*M* = 34.33, SD = 12.52). The analysis showed no effect on the condition factor of patient anxiety *F* (3,135) = 0.077; *p* = 9.72, η^2^*p* = 0.002. Thus, no matter the nature of the condition (contemplative VR, participatory VR, music listening and classical scar massage session), there was a reduction in anxiety after the physiotherapy session.

However, the level of anxiety after the physiotherapy sessions was lower when the patients used contemplative VR (*M* = 26.22, SD = 6.41) and participatory VR (*M* = 27.02, SD = 8.10) than when they listened to music (*M* = 28.41, SD = 8.95) or received a classical scar massage session (*M* = 28.83, SD = 9.59), but there was no statistical significance.

The interaction between the measurement timing and the condition was significant *F* (3,135) = 3.579; *p* < 0.001, η^2^*p* = 0.074. The condition had an effect on the measurement timing. However, according to the *t*-test, no significant difference was observed before or after the experiments.

For our own information, we wanted to know if the anxiety trait could have an influence on the situational anxiety in patients. Data was encoded and transformed according to the guidelines of the *STAI-YB* standard. The French recommendations of *S.T.A.I.* type *Y.B*. (Spielberger et al., 1993) consider that the average on the anxiety trait scale is 45.09 for women. Above this average, they are considered to be anxious. Therefore, we calculated a *t*-test to compare the average anxiety trait in breast cancer patients to the norm in adult women.

The differences between the average of the patients (*M* = 41.72, SD = 10.82) and the norm (*M* = 45.09, SD = 11.11) were significant *t* (45) = −2.113, *p* = 0.040. As *t cal* < *0*, patients did not have an anxious nature. The anxiety that the patients generally felt did not influence the anxiety associated with physiotherapy sessions.

### Cybersickness

The risks of side effects from using virtual reality were analyzed. It appears that participatory VR did not present more risks of cybersickness than contemplative VR *F* (1,45) = 0.09; *p* = 0.64, η^2^*p* = 0.005. The negative effects of VR were not greater during participatory immersion (*M* = 1.44, SD = 0.66) than during contemplative immersion (*M* = 1.51, SD = 0.78). Specifically, only 8.70% of patients experienced mild physical discomfort following VR (4 out of 46).

### Experiment Feedback

**FIGURE 1:**
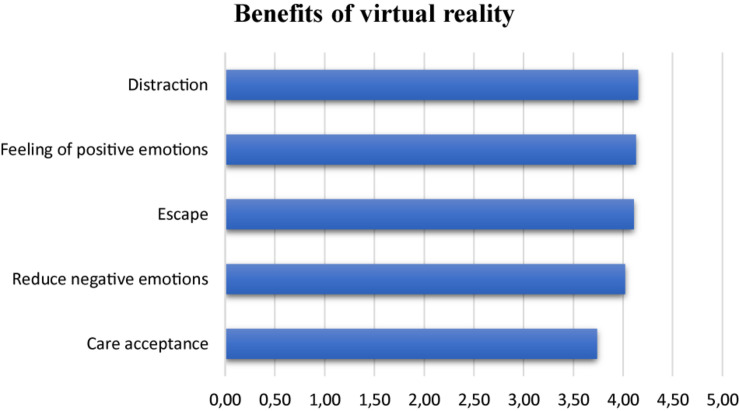
Benefits of virtual reality.

Qualitative data collected from the entire group (*n* = 46) revealed that patients were very supportive of using VR after their immersive experiences (*M* = 8.26 ± 1.31). Very few of them suffered from physical inconvenience following the immersion (*n* = 4, or 8.70%). The majority had the feeling of losing track of time during VR (*n* = 26, or 60.87%). According to the patients’ feelings (see Figure 1), VR offered above all “the possibility of being distracted more easily” (*M* = 4.15 SD = 0.84) and is a good way to regulate emotions: it made it possible to “promote positive emotions” (*M* = 4.13 SD = 0.88) and to “decrease negative emotions and to relax” (*M* = 4.02 SD = 1.04). In addition, it also offered the advantage of “escaping from the medicalized place for a moment” (*M* = 4.11 SD = 1.06) and of better “accepting care” (*M* = 3.74 SD = 1, 16). Regarding the level of immersion, most of the patients preferred to use participatory VR (*n* = 38, or 82.61%). However, it appears that the possibilities of interactions with the environment in the condition of participatory VR were limited: 45.65% of patients (i.e., *n* = 21) considered that the “active” mode was not sufficient. Similarly, 67.39% of patients would have liked to have been offered VR sessions in the form of evolving scenarios (i.e., *n* = 31). Finally, all patients would recommend the VR device during physiotherapy sessions to people with cancer (*M* = 8.02 ± 1.54).

## Discussion

Numerous studies have shown that the distraction power of VR is a fundamental asset in healthcare. Chirico et al. (2019) recently confirmed the benefits of VR in relieving anxiety and improving the emotional state of patients with breast cancer. Like the study by Chirico et al. (2019), we compared the effectiveness of two immersive VR modes (contemplative VR vs. participatory VR) as a distraction tool in the same group of women with breast cancer during physiotherapy rehabilitation sessions.

We started with the hypothesis that the power of distraction from using VR would be all the more effective when it offered the possibility of performing actions in the virtual environment by allowing more attention to be diverted than just passive observation. A so-called participatory immersion would induce a feeling of more intense presence than a contemplative immersion while reducing the anxiety inherent in care situations, thus, improving the emotional state and reducing the perceived time of treatment.

To test these hypotheses, we offered each participant four sessions: participatory immersion, contemplative immersion, music listening and classical scar massage session. We analyzed the feeling of presence in the virtual world (*ITC-SCOPI*), the perception of time as well as the anxiety and emotional state of the patients (*STAI, SAM*) at each of the sessions. We also evaluated the side effects related to VR (cybersickness). Finally, we collected the impressions of patients on the virtual immersions experienced.

### Feeling of Presence and Perception of Time

In view of our results, we can consider that participatory immersion increases the significance of the *feeling of presence* in the virtual universe. In accordance with our hypothesis, we obtain a more intense spatial presence with participatory VR than with contemplative VR. Thus, the mode of immersion can have an influence on the authentic feeling of existing in the virtual environment with the feeling of being immersed in the heart of the scenario.

When it comes to reducing *temporal perception*, VR has proven to be very effective. The significant differences between the real time and the estimated time lead to the conclusion that immersion can help to pass the time faster during physiotherapy sessions than music. However, the analysis does not support the hypothesis that participatory VR decreases the duration perception of physiotherapy sessions more than contemplative VR. Therefore, the two immersions favorably modify the perception of time. The results of our study are consistent with the results reported by Schneider et al. (2011) showing that the treatment seems shorter in patients who use VR compared to those who do not have distraction. According to these authors, the feeling of losing the notion of time could be linked on the feeling of presence in the environment. The relationship between patient temporal perception and the effectiveness of VR distraction has to be furthers documented in future research.

### Mood States

The contributions of VR, according to the questionnaires, enhance the hypothesis of Chirico et al. (2019) stipulating that virtual support is a very good distraction technique. Its main asset is emotional management by increasing positive emotions such as happiness or joy and reducing negative emotions such as anxiety. Based on positive psychology of Fredrickson (2001), the promotion of positive emotions can create and strengthen sustainable and useful personal resources to deal with difficult times (Baños et al., 2013). In addition, whatever the distraction offered, we observed, after the physiotherapy session, an increase in positive emotional state, a feeling of joy and of calmness in the patients. Thus, participatory and contemplative immersions offer patients a moment of escape during physiotherapy sessions that can promote acceptance of care. Contrary to our expectations, the analysis does not confirm the hypothesis that participatory VR is significantly more effective than contemplative VR or music listening in eliciting positive emotion. Surprisingly, we observe a significantly higher sense of joy after the contemplative immersion than after the participatory immersion, while a large majority of patients reported a preference for participatory VR.

The *Self-Assessment Manikin* scale does not identify with certainty what silent emotion lies behind the higher arousal. This increase in the level of arousal in the two VR conditions could represent an experimental artifact since it refers to a form of curiosity and/or excitement about the idea of discovering and experimenting with an innovative technological device (to which patients had not yet been exposed). The instruction of the experiment may have contributed to this result through an announcement effect of this new technology. We can therefore assume that this high arousal level would tend to disappear with repeated exposure to the VR device.

### Anxiety

Our results show a significant decrease in anxiety in all patients after the treatment session. All the patients showed a decrease in anxiety after the physiotherapy sessions whatever the distractions offered (participatory VR, contemplative VR, and music listening), and also during the physiotherapy session without intervention other than that of the practitioner present at their sides (control condition). While the anxiety level was lowest under contemplative VR, it did not significantly differ from the anxiety levels observed under the other three conditions.

Chirico et al. (2019) used the stress and adaptation model of Lazarus and Folkman (1984) that define coping as the set of cognitive and behavioral efforts to control, reduce or tolerate aversive situation (Lazarus and Folkman, 1984). VR distraction would be an active “vigilant” strategy. Patients would regulate the emotional response associated with stressful medical procedures through selective attention they focus on the pleasant stimuli in VR distraction. Unlike the results of Chirico et al. (2019), our study shows lower anxiety in patients after physiotherapy sessions whatever the support methods. Our results do not allow the drop in anxiety level to be attributed to virtual reality or music listening, which unexpectedly underlines the importance of the practitioner’s empathetic presence alongside patients. Compared to Chirico et al. (2019), the care context is different and, in fact, our conditions are not comparable with each other. Indeed, if in chemotherapy the patients are most often alone for the duration of the treatment, in physiotherapy, the practitioner remains involved throughout the treatment phase alongside the patients. Therefore, the presence of the practitioner and his interactions with the patient provide a context that is just as favorable in reducing anxiety as the emotional regulation tools used (VR, music listening). In addition, not all patients appreciate being distracted during disaggregated medical treatments, some may prefer to maintain a sense of control and observe the routine of care. According to Garrett et al. (2020), VR is a powerful distractor whose effectiveness would depend on visual, sound, cognitive and emotional engagement, as well as personal acceptance of this technology. Future research would benefit from evaluating patients’ appreciation, motivation, and ability to process VR sensorimotor information to determine their degree of involvement in distraction.

If the clinical conditions create a calming climate (in favor of reducing anxiety), the use of technological tools could be favored when the practitioner is not available during the treatment phase or, again, to reduce the monotonous nature of repetitive therapy sessions.

### Cybersickness

Regarding the risk of side effects in an immersive situation, we do not see any significant negative effect of VR, whatever the methods of immersion. No significant physical inconvenience such as nausea, headache, dizziness or eyestrain was reported in patients who participated to the whole study. These results are in line with Chirico et al. (2019) who reported negligible and infrequent side effects. The same is true for first time users of VR in palliative care, showing that no patient complained of serious discomfort related to VR travel (Niki et al., 2019). It should be noted that the risks of cyber-malaises are now easier to avoid with new generation VR device as like the one used in this experiment.

### Experiment Feedback

In view of our questionnaire, the interest in VR is obvious. Patients are very favorable about using it without feeling any physical inconvenience to the device. Our results allow us to define the contributions of VR and the preferences of immersion in women with breast cancer in post-surgical care.

First, VR experience should be viewed in light of the benefits that patients give them. The therapeutic benefits of VR are mainly associated with its distractive power, which facilitates the emotion management. Patients have the feeling of escaping reality and of losing track of time in a soothing environment. VR also contributes to care acceptance with an equally positive assessment of its advantages during post-surgical physiotherapy sessions.

Secondly, the immersion offered to patients must be interactive enough to maintain patient interest in the virtual environment. The constantly renewed technology should allow, in the near future, the use of software allowing the patient to engage more in the immersive task, by mobilizing her cognitive resources ranging from simple distraction to concentration or skill reinforcement (Ahmadpour et al., 2020) through evolving scenarios.

Thirdly, VR should be considered at different stages of cancer management. Based on our quantitative data, patients seem to recommend the use of this device in the context of post-surgical physiotherapy rehabilitation.

### Limitations

Like all experimental research, this study has certain limitations. As Chirico et al. (2019) pointed out, it would be prudent to have physiological measurements in order to compare them with the subjective results obtained from questionnaires. Because our results are based primarily on declarative measures, a desirability bias could have influenced the responses of our patients. Future research would benefit from setting up, in addition to the questionnaires, an electrodermal recording in order to obtain a more objective measurement of the effect of VR on emotional state.

In addition, like Chirico et al. (2019), it would be desirable to offer a familiarization phase for the use of VR before starting the actual experiment. This preliminary phase would reduce the surprise effect and the naive attractiveness for a more accurate measurement of emotional states associated with the use of VR. Studies that have implemented a familiarization phase in their research protocol have all observed significant results in reducing anger, pain, anxiety or symptom distress (Schneider and Workman, 1999; Gershon et al., 2004; Schneider et al., 2004; Schneider and Hood, 2007; Atzori et al., 2018; Chirico et al., 2019; Tennant et al., 2020). Therefore, we can assume that there is a link between familiarization and the significant results obtained during the immersive experience.

According to our results, participatory immersion induces a feeling of spatial presence in the virtual world that is more intense than contemplative immersion, without increasing the patients’ engagement in this interactive task. Make no mistake, motor mobilization alone does not guarantee better patient engagement in the immersive environment, as a gradual cognitive component is necessarily associated with it. Like Ahmadpour et al. (2020), the potential actions offered by VR must be cognitively stimulating in order to engage the patient and promote the emergence of a feeling of presence. The lack of difference in our study between the two immersive modes raises the question of the effectiveness of cognitive stimulation made in the participatory environment. According to Bouvier (2009), the effectiveness of VR is evaluated positively when many patterns of interactions between the user and the virtual system are offered by the interface. We can assume that the new generation of VR systems, more interactive, mobilizing more cognitive resources, will be able to reinforce the benefits of the power of distraction and be more effective in relieving the anxiety associated with cancer.

Because the existing devices are not sufficiently thought out to couple the action-cognition modality, repeated exposure risks causing a habituation phenomenon that is detrimental to the effectiveness of the device in terms of emotional management. In summary, VR should be more flexible in terms of cognitive stimulation and provide a wider range of emotional variations, tailored to the patient’s needs at the time *t*, in order to create a more personalized tool. In other words, the variability of the scenarios, the subject-environment interaction and its dynamics could reinforce the distraction by maintaining the attention of the patients in an environment which would be their own and which they would constitute according to a progressive and singular real/virtual coevolution (de Loor and Tisseau, 2011).

These perspectives of research and development of virtual reality tools will gain by being anchored in the theoretical conceptions of embodied and situated cognition (Versace et al., 2018) where the VR device stimulates the individual at the sensorimotor as well as the cognitive level for a more effective impact on the emotional level.

## Data Availability Statement

The original contributions presented in the study are included in the article/supplementary material, further inquiries can be directed to the corresponding author.

## Ethics Statement

Ethical review and approval was not required for the study on human participants in accordance with the local legislation and institutional requirements. The patients/participants provided their written informed consent to participate in this study.

## Author Contributions

All authors listed have made a substantial, direct and intellectual contribution to the work, and approved it for publication.

## Conflict of Interest

The authors declare that the research was conducted in the absence of any commercial or financial relationships that could be construed as a potential conflict of interest.
